# The Importance of Microbiota and Fecal Microbiota Transplantation in Pancreatic Disorders

**DOI:** 10.3390/diagnostics14090861

**Published:** 2024-04-23

**Authors:** Adrian Boicean, Cristian Ichim, Samuel Bogdan Todor, Paula Anderco, Mirela Livia Popa

**Affiliations:** Faculty of Medicine, Lucian Blaga University of Sibiu, 550169 Sibiu, Romania; adrian.boicean@ulbsibiu.ro (A.B.); samuelbogdant@gmail.com (S.B.T.); paula.anderco@ulbsibiu.ro (P.A.); liviamirelapopa@yahoo.com (M.L.P.)

**Keywords:** microbiota, fecal microbiota transplantation, pancreatitis, pancreatic cancer, T1D

## Abstract

The role of the intestinal microbiota in the diagnosis and treatment of pancreatic diseases is increasingly significant. Consequently, fecal microbiota transplantation (FMT) is emerging as a promising therapeutic avenue for various pancreatic disorders, including cancer, pancreatitis, and type 1 diabetes (T1D). This innovative procedure entails transferring gut microbiota from healthy donors to individuals affected by pancreatic ailments with the potential to restore intestinal balance and alleviate associated symptoms. FMT represents a pioneering approach to improve patient outcomes in pancreatic diseases, offering tailored treatments customized to individual microbiomes and specific conditions. Recent research highlights the therapeutic benefits of targeting the gut microbiota for personalized interventions in pancreatic disorders. However, a comprehensive understanding of the intricate interplay between gut microbiota and pancreatic physiology warrants further investigation. The necessity for additional studies and research endeavors remains crucial, especially in elucidating both adult and pediatric cases affected by pathological pancreatic conditions.

## 1. Introduction

The medical community is increasingly focusing on the intestinal microbiota, especially due to its discovered links to the functioning of various organs in the human body [1]. Microbiota alterations seem to be especially beneficial for diagnosing certain pancreatic pathologies and it is possible that the future of non-invasive diagnosis may emerge from this realm [2]. Certainly, influencing the microbiota through fecal microbiota transplantation (FMT) is gaining recognition as a promising treatment strategy for a range of conditions, including pancreatic diseases like cancer, pancreatitis, and diabetes. FMT involves transferring gut microbiota and their byproducts from an individual in good health to one who is ailing. This procedure is drawing attention for its effectiveness and the ease of use, presently considered the most effective method for restoring intestinal flora and addressing both intestinal and potentially non-intestinal illnesses [3,4,5]. This technique involves altering the composition of the gut microbiota to normalize it, thereby obtaining therapeutic benefits [6,7].

As Antushevich outlines, the selection of donors for fecal material is subject to very stringent criteria to mitigate the risk of disease transmission from a nominally healthy donor to the recipient [8]. Before undergoing FMT, donors are subjected to thorough screening to rule out conditions such as HIV, syphilis, hepatitis A, B, C, or autoimmune diseases. Similarly, donors must not be overweight and they must be free of any tumors, inflammation, diabetes, infectious diseases, or metabolic syndromes [8,9,10,11].

Up until 2020, the only established application for FMT was in treating the persistent and difficult cases of the *Clostridioides difficile* infection (CDI), where it has been shown to be highly effective, achieving success rates of over 80–85% [12,13,14]. Recent studies, however, indicate that disorders specific to the pancreas might be influenced by the microbiota, whether through the direct effects of the pancreatic microbiota or indirectly via the intestinal microbiota [15,16,17].

The link between such microbial modulation and the emergence of pancreatic diseases, including pancreatic ductal adenocarcinoma, acute and chronic pancreatitis, and type 1 diabetes mellitus, will be explored. This insight opens up the potential use of the FMT in the treatment of these pancreatic conditions. This review aims to collate and discuss recent research on how gut microbiota can be utilized in diagnosing pancreatic pathologies through distinctive changes and particularly how modulating the microbiota via FMT can impact treatments for various pancreatic disorders, highlighting its potential benefits.

## 2. Microbiota Changes in Pancreatic Disorders

Analyzing microbiota is increasingly widespread in the study of digestive tract disorders, including those affecting the pancreas. Several authors have started identifying specific microbiota patterns for each disease. This approach could markedly enhance the diagnosis of future patients, especially in the cases of pancreatic cancer, by relying exclusively on the analysis of the microbiota [18,19]. Microbiota analysis is non-invasive, which could increase patient compliance with this type of testing and consequently increase the number of diagnoses at an earlier stage. Ren et al. not only showed that certain bacterial species are found in higher quantities in patients suffering from a certain pathology, but also observed notable differences in the quantity of *Streptococcus* in stool between those with pancreatic head cancer and those with pancreatic body cancer. The interaction between the intrapancreatic microbiome and gut microbiota has been recognized as a factor affecting the progression of patients with pancreatic tumor diseases [20]. Lu et al. have even demonstrated that the pattern of oral microbiota alterations can be correlated with the diagnosis of pancreatic cancer [21]. Both oral and rectal microbiota were utilized in a multicentric study involving over 400 patients, which identified that the composition of the microbiota can predict the course of acute pancreatitis. In the future, this kind of analysis could play a role in diagnosing pancreatitis and in directing treatment strategies [22].

## 3. Fecal Microbiota Transplantation (FMT) and Its Role in Pancreatic Illness

It is important to understand the foundational techniques involved in preparing the FMT, which may differ depending on the illness being addressed. The approach to donor selection, screening, and fecal material processing must be customized to suit the condition at hand [23].

**Figure 1:**
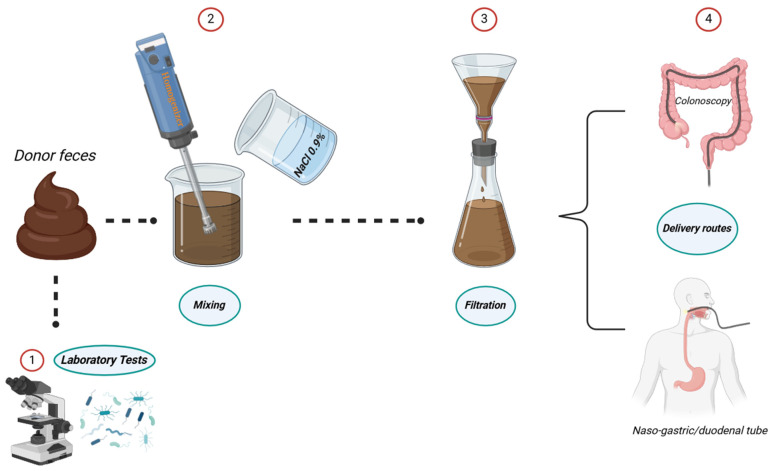
Necessary steps for preparing the stool for microbiota transfer.

A procedure for FMT, particularly aimed at treating CDI, is detailed by Perez et al. [24]. It involves mixing 50–60 g of stool with 200–300 mL of a diluent until it becomes a liquid suspension [24]. After allowing the mixture to settle for 5 min, it is filtered through gauze. The resulting liquid can be used immediately, refrigerated for up to 24 h at 2–8 °C, or frozen for up to 30 days at −20 °C. If glycerin is added, the fecal material can be preserved at −80 °C without losing its effectiveness. The method of FMT administration can vary, including oral (via the upper gastrointestinal tract), nasal, or rectal (via colonoscopy) routes [25,26,27]. Rectal administration is often preferred due to the fecal substrate’s location deep within the cecum, minimizing the risk of its removal (Figure 1). However, administering FMT through a nasogastric or nasoduodenal tube may lead to complications from the high levels of pathogenic bacteria in the upper digestive and respiratory tracts, potentially causing pulmonary or gastrointestinal issues [24]. Important studies in the literature have underscored the feasibility of culturing the microbiota to manipulate its content, aiming to stimulate the growth of specific bacterial strains, thus leading to the enhanced efficacy of the FMT [28,29].

In addition to its role in combatting recurrent CDI, FMT has shown promise in the field of oncology, especially in mitigating the side effects of tumor radiotherapy [30,31,32]. Radiation therapy often leads to damage and has negative effects on the gut microbiota’s composition [33]. Research by Cui et al. highlighted that animals subjected to radiation exhibited a higher survival rate following FMT [34]. Furthermore, both male and female mice that received the transplant showed an increase in peripheral white blood cell counts, along with enhancements in gastrointestinal function and the integrity of the intestinal epithelium [34]. Consequently, the researchers proposed that FMT could serve as a radioprotective agent, potentially improving outcomes in cancer treatment involving radiotherapy [34,35].

In addition to the discoveries concerning radiotherapy, a groundbreaking development in oncology involving the gut microbiota is the realization that the pancreas possesses its own unique microbial makeup. This challenges the previous belief that the pancreas was a sterile organ [15,36]. Numerous studies have confirmed the presence of micro-organisms within the pancreas under non-diseased conditions, referred to as the inherent pancreatic microbiota [16]. Although research in this area is still in its early stages and thus limited, this revelation introduces new avenues for exploring oncogenesis and identifying novel treatment targets for pancreatic tumors.

Additionally, a novel concept known as the “microbiota-pancreas axis” has been introduced, detailing a bidirectional communication system that illustrates the influence of pancreatic physiological functions on the intestinal microbiota and how the intestinal microbiota, in turn, affects the pancreas [37,38,39]. The FMT induces a notable response, particularly involving regulatory T cells, iNKT cells, and Antigen Presenting Cells (among others), which diminish the inflammatory response. This has been evidenced not only in patients with *Clostridioides difficile* infection but also in those with other pathologies. Reducing the activity of the inflammatory system could offer substantial advantages in pancreatic conditions, a significant number of which are inflammation-driven [40,41,42]. Building on this, the discussion will extend to the effects of both the inherent pancreatic and intestinal microbiota on the progression of intrinsic pancreatic diseases, whether malignant or benign.

The bacteria identified in the transplanted fecal matter that showed the most significant beneficial effects for patients with various pathologies are listed in Table 1, but the table also highlights the dominant microbiotas in humans and mice. These are particularly important because they can be stimulated to enhance the desired effect of the FMT.

## 4. The Intestinal-Pancreatic Axis

The gut–pancreatic axis, modulated by the microbiome, plays a pivotal role in pancreatic immunity and disease pathogenesis. This axis is defined by critical interactions: gut-derived short-chain fatty acids (SCFAs) regulate immune responses in the pancreas by controlling the production of the cathelicidin-related antimicrobial peptide in beta-cells [50]. Furthermore, pancreatic acinar cells affect gut microbiota and intestinal immunity through the secretion of specific antimicrobials [50]. These findings refute the traditional view of the pancreas as a sterile environment, illustrating instead a dynamic pancreatic microbiome of migratory bacteria and fungi from the gut, which may impact diseases like pancreatic ductal adenocarcinoma (PDAC) [50].

## 5. Pancreatic Cancer

Pancreatic cancer (PC) ranks among the deadliest diseases globally. In the United States, pancreatic ductal adenocarcinoma (PDAC), a particular type of pancreatic tumor, is recognized as the third highest cause of cancer death and accounts for 85% of all malignant pancreatic conditions [51].

The combination of ineffective treatments and late diagnosis contributes to the extremely low survival rates in pancreatic cancer, further hampered by the slim prospects for successful tumor removal. Although the complex interactions between pancreatic cancer and the gut microbiota are recognized, it is still unclear whether a causal relationship exists. Modifying the gut microbiota presents a promising approach for influencing tumorigenesis and the management of pancreatic cancer in the future, with ongoing research exploring antibiotics, probiotics, and FMT as potential treatments [52,53,54].

Studies have indicated that individuals with periodontal disease exhibit a higher incidence of PDAC compared to those without periodontal issues [55]. In light of this, Farrell and colleagues’ study was conducted to profile the oral microbiome of patients to explore its potential link with pancreatic cancer [56]. The researchers identified significant microbiome differences using bacterial microarrays and qPCR validation. Specifically, PDAC patients showed alterations in 31 species and reductions in 25, notably within the *Firmicutes*, *Proteobacteria*, and *Actinobacteria* groups. Further validation highlighted *Neisseria elongata* and *Streptococcus mitis* as significantly reduced in PDAC patients, suggesting their potential as biomarkers for the disease [56].

Pushalkar et al. undertook a study to investigate if gut bacteria could move to the pancreas, employing *Enterococcus faecalis* marked with fluorescent labels and *Escherichia coli* tagged with GFP, administered to mice [57]. The study confirmed bacterial migration to the pancreas, suggesting a direct impact of the gut bacteria on the pancreatic environment. Further, using 16S rRNA FISH and qPCR, a higher bacterial abundance was found in PDAC in both mice and humans compared to normal pancreas. The sequencing of 16S rRNA genes in human PDAC tumors identified 13 bacterial phyla with Proteobacteria, Bacteroidetes, and Firmicutes being predominant. Significant differences in bacterial composition between PDAC and normal pancreas were highlighted and oral antibiotics were found to slow oncogenesis, while transferring specific bacteria or fecal material from PDAC mice accelerated tumorigenesis. These results underline the microbiome’s role in disease progression and suggest that targeting the microbiota could potentially reduce PDAC risk [57].

FMT is being recognized as a promising strategy that could hinder the progression of pancreatic cancer. This process might be enabled by adjusting the gut microbiota, which includes decreasing the production of the inflammatory agents and cytotoxic byproducts, as well as correcting dysbiosis in the gut flora [58].

Another research focused on FMT related to pancreatic cancer involved transferring fecal matter from subjects with advanced stages of PC, those who have survived PC for more than five years, and healthy participants into mice. Thirty-five days after the transplantation, mice that received the FMT from the group with advanced pancreatic cancer showed markedly larger tumors compared to those receiving FMT from long-term PC survivors or healthy controls [20].

In addition to the previous studies about the connection between periodontal disease and a higher risk of PC, Castillo et al. explored the link between oral bacteria and pancreatic cancer progression by examining tissue samples from 50 pancreatic cancer patients at Rhode Island Hospital and 34 organs from the National Disease Research Interchange [59]. Using 16S rRNA gene sequencing on 189 tissue samples and 57 swabs, along with 12 stool samples, the study found diverse bacterial DNA in pancreatic tissues [59]. Bacterial DNA varied highly among individuals and sites within the pancreas and duodenum, regardless of cancer presence. Non-cancer subjects had higher levels of Lactobacillus, while cancer subjects showed an increased abundance of the *Fusobacterium* spp., known to be associated with colorectal cancer [59]. This study suggests bacteria may migrate from the gut to the pancreas, highlighting the need for further research on their potential causal role in pancreatic cancer [59].

Mitsuhashi et al. found that individuals with PC exhibit a higher abundance of Fusobacterium compared to healthy individuals. Their research further indicated that the increased occurrence of the *Fusobacterium* species in tissues affected by pancreatic cancer independently correlates with a worse outcome. This suggests that *Fusobacterium* species might serve as a promising prognostic marker for pancreatic cancer [60].

These studies collectively suggest that both intra- and extra-pancreatic microbiota play roles in the development, progression, and severity of pancreatic cancer, paving the way for potential new treatments centered around microbiota. FMT, known for its effectiveness and safety in conditions like *Clostridioides difficile*, emerges as a promising option. However, research on FMT’s use in pancreatic cancer treatment is sparse. Upcoming studies on animals are essential to verify the effectiveness and safety of the FMT and to investigate its potential for clinical application. There is also a need for tailored therapeutic strategies that target specific gut microbiota functions, improve transplant compatibility, and refine personalized FMT treatments for pancreatic cancer [3].

## 6. Acute and Chronic Pancreatitis

In addition to PC, other pancreatic disorders like pancreatitis, which can be either acute or chronic, also exhibit changes in gut microbiota composition, highlighting them as potential targets for FMT treatments.

Acute pancreatitis (AP) is a medical emergency characterized by the rapid-onset inflammation of the pancreas that can escalate into a systemic illness, presenting symptoms such as intense abdominal pain, vomiting, and nausea and may result in serious complications like infection and organ failure [61,62,63]. Given the critical nature of AP and its potential to endanger life, understanding the microbiome’s influence on the disease’s progression and severity has become a key research focus. This includes investigating how dysbiosis or microbial imbalance in the gut flora may contribute to the breakdown of the intestinal barrier function [61].

Patients with AP have been found to show changes in the diversity and composition of their intestinal microbiota [64,65,66,67,68]. Research indicates that acute inflammation can heighten intestinal permeability, possibly by altering the expression of claudin-4, a protein vital for tight junction integrity in intestinal epithelia [69]. Reduced claudin-4 levels weaken the connections between epithelial cells, impairing the epithelial barrier and resulting in an increased permeability. This allows substances to pass through more easily, potentially causing systemic and pancreatic bacterial translocation and perpetuating inflammation [69].

In addition to the increased permeability caused by inflammation, other contributing pathological mechanisms, such as microcirculation changes and ischemia–reperfusion injury also induce similar effects [37]. These changes compromise intestinal permeability, resulting in a condition referred to as leaky gut. When coupled with bacterial overgrowth, this leaky gut condition further facilitates the movement of bacteria and toxins towards the pancreas, aggravating pancreatic inflammation. This escalation of the inflammation can lead to further damage, potentially resulting in fibrosis or, in more severe instances, necrosis [37].

Furthermore, recent research has observed a rise in pathogenic bacteria during acute inflammatory states, alongside a reduction in short-chain fatty acids (SCFAs) such as propionate, acetate, and butyrate, which are metabolites generated by the gut bacteria via the fermentation of dietary fibers [20]. They play a vital role in maintaining gut homeostasis by restoring its flora, strengthening the intestinal epithelial barrier, and modulating inflammation [61]. They can also reduce systemic inflammatory responses, aid in the repair of the damaged pancreas and prevent dysfunction in other organs. Given these multiple benefits, increasing SCFA levels could represent a novel protective strategy for treating AP. Such treatments could be directly applied through butyrate administration or indirectly through fiber and probiotic supplementation via FMT. These approaches offer promising additions to conventional enteral nutrition support in AP therapy.

A stratified study analyzing patients with AP found that microbiota alterations vary with the disease’s severity, notably including a decrease in SCFA-producing bacteria which correlates with increased severity due to compromised intestinal barrier integrity [70]. Zhu et al. collected clinical data and fecal samples from 165 adults, revealing that severe AP is marked by a reduction in commensal bacteria like Bacteroides, Alloprevotella, and Blautia [70]. Further investigation in male mice with AP showed significant differences in intestinal microbiota compared to healthy controls, linking altered microbiota with systemic inflammation and intestinal barrier dysfunction.

Tan et al.’s findings align with Zhu et al.’s research, highlighting that microbial composition changes as AP progresses [71]. Specifically, they observed a significant rise in potentially pathogenic bacteria like Enterobacteriaceae and Enterococcus in severe AP (SAP) compared to milder forms (MAP or MSAP) [71]. Both studies conclude that the intestinal microbiota significantly influences AP as a mediator, with its imbalance associated with the disease’s severity [71].

Yang et al. documented a case where FMT was applied to a patient with moderately severe acute pancreatitis (MSAP) complicated by a severe CDI, a condition for which FMT is a common treatment [72]. The treatment led to the resolution of diarrhea within five days, with no adverse events reported and a colonoscopy 40 days after discharge showed complete recovery. However, the impact of the FMT on MSAP itself was not evaluated and while some studies have explored FMT in AP within mice models, showing increased bacteria translocation and mortality in AP mice receiving FMT from healthy ones, the effects and safety of the FMT for AP in humans remain unclear, indicating a need for further research [72].

Chronic pancreatitis (CP) is defined by the persistent inflammation of the gland, marked by distinctive features including the dilation of the Wirsung duct, calcifications, atrophy, and fibrosis [73]. Chronic pancreatitis results in the progressive dysfunction of both the endocrine and exocrine systems, with studies showing that individuals with CP undergo dysbiosis characterized by a rise in pathogenic bacteria [74,75].

Pan et al. highlight that intestinal dysbiosis, especially the reduction in SCFA-producing bacteria, accelerates the advancement of CP, similar to what is observed in AP [70,76]. Reduced SCFA levels undermine intestinal barrier integrity, leading to worsened pancreatic fibrosis, increased monocyte recruitment, and enhanced M2 macrophage polarization [77]. Supplementation with SCFAs has been shown to bolster intestinal barrier function and decrease monocyte recruitment, thereby offering greater protection against CP development. This highlights the therapeutic potential of dietary SCFAs and the targeting of SCFA-producing Gram-positive bacteria in CP’s prevention and management [77]. Additionally, studies indicate that pancreatic enzyme replacement therapy (PERT) effectively manages exocrine pancreatic insufficiency (PEI) symptoms by improving patients’ nutritional status [77,78].

Nishiyama et al. analyzed fecal samples from mice receiving pancreatic enzyme replacement therapy (PERT) compared to control mice to investigate changes in the intestinal microbiota. Their results bolstered the theory that PERT alleviates symptoms associated with exocrine pancreatic insufficiency (PEI), observing modifications in the gut microbiota composition of the treated mice. In particular, mice treated with PERT exhibited a notable rise in *Akkermansia muciniphila* and *Lactobacillus reuteri* populations [79].

In a double-blind, controlled, randomized trial, Dos Santos and colleagues examined the impact of synbiotics on the gut microenvironment in patients with CP [80]. The intervention group was administered a synbiotic combination that included 12 g daily of Lactobacillus casei, Lactobacillus rhamnosus, Lactobacillus acidophilus, and Bifidobacterium bifidum, whereas the control group was given 12 g daily of a medium absorption complex carbohydrate [80]. The research concluded that synbiotics resulted in better clinical and laboratory results for patients with CP, highlighting the potential of strategies aimed at manipulating the intestinal microbiome as viable treatments for this chronic condition [80].

These studies collectively hint at microbiota manipulation, with its affordability and ease of implementation, as not only a novel therapeutic avenue for CP but also as a preventive strategy to mitigate its symptoms and complications.

## 7. Type 1 Diabetes

Type 1 diabetes (T1D) is an autoimmune disorder characterized by the autoimmune destruction of the β cells in the pancreas, which are responsible for insulin production, and is another disorder likely linked to the dysregulation of our microbiota. Consequently, T1D may also stand to benefit from FMT treatments [74,81,82,83]. The development of T1D is shaped by environmental and genetic influences that affect immune regulation, with factors like viral infections, diet, and vitamin D deficiency linked to its onset.

Research, including a 2018 longitudinal study, has explored how gut microbiota may predispose individuals to T1D [84]. This study tracked the gut microbiomes of infants at genetic risk for T1D, collecting fecal samples from 3 months old until T1D diagnosis. Although the overall microbial composition was similar between T1D cases and controls, children without T1D showed a higher abundance of SCFA-producing bacteria in their gut microbiomes.

The research suggests that SCFAs may play a protective role in preventing T1D, as a lower presence of SCFA-producing bacteria is noted in T1D cases [74]. Zheng et al. also found a link between T1D and an increased *Bacteroidetes/Firmicutes* ratio, along with a reduced α-diversity in fecal microbiota [85]. T1D individuals showed higher levels of certain bacteria like *Clostridium* and *Bacteroides*, while beneficial bacteria such as *Lactobacillus* and *Bifidobacterium* were less common. This imbalance in gut microbiota is associated with an increased intestinal permeability seen in T1D children. Although no current treatments directly target gut microbiome alterations to delay or prevent T1D, emerging data point towards potential strategies, including microbiome modulation and enhancing diversity, perhaps by using *Escherichia coli* Nissle (EcN) to reduce pathogenic bacteria colonization [86].

## 8. Therapeutic Prospects in Pancreatic Disorders

### 8.1. Pancreatic Cancer

The role of micro-organisms like HCV, HBV, Helicobacter pylori, and HPV in cancer development highlights a complex interplay between environmental factors and host genetics, suggesting micro-organisms may synergistically contribute to tumorigenesis. The microbiota, a community of such micro-organisms, has been recognized as a crucial element in cancer biology. Although not fully understood, the interaction between the microbiota and cancer may involve bacterial metabolites that offer protective benefits against tumors [81]. For instance, acetate not only mitigates pancreatitis and reduces PDAC risk factors but also influences epigenetic changes in mesenchymal stem cells, promoting their transformation into cancer-associated fibroblasts that increase PDAC cell invasiveness. The link between the microbiota and pancreatic cancer was initially proposed following the detection of *H. pylori* (HP) in pancreatitis patients, leading to further research on the connections between fecal, pancreatic, intestinal, and oral microbiota and pancreatic cancer [81].

Xu’s study finds a significant link between HP infection and pancreatic cancer, especially in economically underdeveloped regions. However, no positive association is observed between specific H. pylori strains (CagA+ or VacA-positive) and pancreatic cancer [87]. While HP infection overall may raise pancreatic cancer risk, further research is needed to understand this relationship fully [87].

As the literature also underscores, the potential of the FMT as a novel therapeutic avenue is gaining attention. Research suggests a link between gut dysbiosis and pancreatic cancer, potentially due to bacterial translocation [88]. The role of both the oral and endogenous pancreatic microbiota further highlights the intricate microbial interactions involved in pancreatic cancer, underscoring FMT’s potential to address these imbalances. Yet, the efficacy and safety of the FMT in this context require further investigation [53,89].

### 8.2. Acute and Chronic Pancreatitis

AP leads to an imbalance in the intestinal microbiota, exacerbating pancreatic harm and systemic inflammatory reactions. In this context, FMT offers a promising approach to diminish tissue damage and inflammation and to mitigate the dysbiosis [50,90].

In their study, Li Wei Liu and colleagues investigated how the gut microbiota and their metabolites affect AP to understand the related pancreatic damage and inflammation better [91]. They discovered that normobiotic FMT corrects gut dysbiosis caused by AP, reducing its severity, including mitochondrial dysfunction, oxidative stress, and inflammation [91]. This improvement was linked to the increased levels of the NAD+-related metabolites, notably NMN, which mitigated the adverse effects of AP by enhancing pancreatic NAD+ levels. Furthermore, the study highlighted that activating the SIRT3-PRDX5 pathway through normobiotic FMT and NMN metabolism played a crucial role in exerting antioxidant and anti-inflammatory actions, suggesting normobiotic FMT as a viable treatment for PsA [91].

In Ding and his team’s study, they assessed FMT’s role in managing AP, focusing on its effect on intra-abdominal pressure, gastrointestinal function and infection rates [92]. Sixty patients were randomly assigned to receive either FMT or saline, showing no significant difference in gastrointestinal recovery between the two groups [92]. Although FMT led to increased levels of D-lactate and IL-6, suggesting potential adverse effects on the gastrointestinal barrier, it did not significantly benefit PAH or reduce infectious complications [92]. The findings indicate a need for further research to understand FMT’s effects and suggest using changes in gut microbiota as biomarkers for pancreatic fibrosis evaluation.

In the realm of pancreatic inflammation, including acute and chronic pancreatitis, FMT emerges as a promising treatment for addressing dysbiosis and its consequent effects. While its potential to alleviate the severity of acute pancreatitis through the modulation of the gut microbiota and the enhancement of the anti-inflammatory and antioxidative processes is recognized, the therapeutic impact on chronic pancreatitis and the prevention of fibrosis through microbiota modification also warrants further exploration [93,94].

These insights pave the way for new therapeutic strategies for acute, chronic, and autoimmune pancreatitis, with FMT offering a potential means to alter disease progression.

A study conducted by Li et al. explores how saikosaponin A affects the gut microbiota and SAP [95]. Through 16S rRNA gene sequencing and analyzing inflammatory and antioxidant markers, it was found that saikosaponin A promotes a healthier gut, increasing *Lactobacillus* and *Prevotella*, and reduces SAP symptoms [95]. This includes lower serum amylase, lipase, oxidative stress, and inflammation, with a boost in antioxidant signaling (Keap1-Nrf2-ARE) [95]. Similar outcomes from FMT suggest saikosaponin A‘s beneficial effects might be through microbiota improvement, highlighting the need for further investigation [95].

### 8.3. Type 1 Diabetes

T1D often leads to vascular and neurological complications, with current effective treatments being limited to lifestyle changes and pharmacological methods like insulin injections. However, chronic insulin use can result in obesity, hypoglycemia, hyperinsulinemia, as well as psychological and financial burdens. Given that T1D usually affects individuals at a young age and taking into account the significance of the gut microbiota in the disease’s development and progression, FMT presents a promising alternative.

In a 2022 experimental study, He et al. administered one to three cycles of FMT to two adolescent T1D patients, monitoring clinical outcomes, biochemical indices, and adjustments in therapeutic regimen and dosage [81]. The study, supported by the metagenomic sequencing of the fecal microbiota post-transplant, suggests that FMT protocols could be optimized for more effective outcomes, highlighting FMT’s potential as a viable treatment for autoimmune T1D, with implications for improving patient quality of life [81].

De Groot and colleagues found that FMT can preserve endogenous insulin production in patients with T1D diagnosed within the last 12 months [96]. The study divided participants aged 18 to 30, with recent T1D onset, into groups receiving either autologous or allogeneic FMTs for four months [96]. Results showed significant preservation of beta-cell function in the autologous FMT group over 12 months, linked to specific plasma metabolites and inversely related to *Prevotella* in the small intestine [96]. This preservation was evident regardless of the donor type, indicating FMT’s potential role in early T1D management [96].

Table 2 summarizes the main articles in the literature analyzing fecal microbiota transplantation in pancreatic disorders.

For T1D, the gut microbiome’s role in immune modulation suggests that FMT could offer therapeutic benefits by restoring gut microbiome balance, potentially slowing disease progression and reducing inflammation. While preliminary findings are promising, further studies are essential to validate FMT’s safety and efficacy in T1D management and its broader implications on patient quality of life and disease management [97,98].

**Figure 2:**
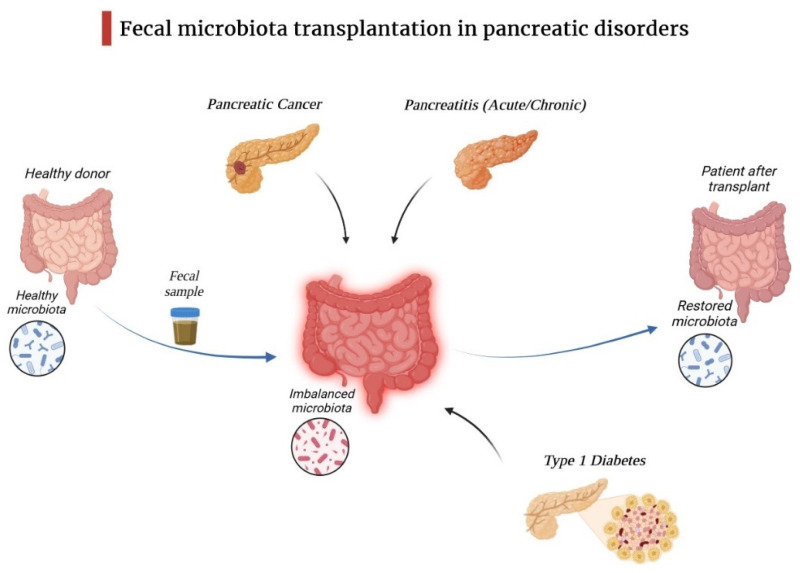
Fecal microbiota transplantation in pancreatic disorders.

Figure 2 highlights the ability of fecal matter transfer to restore microbiota balance in pancreatic pathologies.

Although FMT is generally regarded as safe with few adverse effects, its long-term implications are yet to be thoroughly investigated. Future considerations include determining the frequency and duration of follow-ups post-FMT to monitor for long-term adverse events. The goal moving forward is to tailor FMT treatments to individual patients and specific conditions, considering the diversity of hosts and diseases [99].

## 9. Conclusions

The gut microbiota, a vast and intricate ecosystem, has been identified as a key player in diagnosing pancreatic disorders, but especially in influencing the onset and progression of pancreatic cancer, as well as acute and chronic pancreatitis and type 1 diabetes mellitus. Research into the gut microbiota has unveiled mechanisms underlying these pancreatic conditions, offering insights into their risk, severity, and the potential for new diagnostic and prognostic strategies.

The role of the intestinal microecology in the pancreas and broader physiological processes holds substantial scientific interest, warranting further investigation. The pursuit of novel therapeutic avenues to enhance patient outcomes for pancreatic disorders is crucial, with the microbiota presenting a vast potential for personalized treatments tailored to each individual’s microbiome and specific condition.

## Figures and Tables

**Table 1:** Predominant beneficial bacteria in fecal microbiota transplantation and dominant microbiota in humans and mice.

Dominant Microbiota in Humans and Mice			
Authors	Dominant microbiota	Host	
Li et al. [43]	*Lactobacillus reuteri*, *Enterococcus faecium*, *Escherichia coli*, *Bacteroides ovatus*, *Fusobacterium gastrosuis*	Mice	
Rinninella et al. [44]	*Fecalibacterium prausnitzii*, *Clostridium* spp., *Lactobacillus reuteri*, *Enterococcus faecium*, *Bacteroides vulgatus*, *Bacteroides uniformis Prevotella* spp., *Parabacteroides distasonis*	Human	
**Predominantly Beneficial Microbiota in Various Pathologies**			
Authors	Micro-organism	Pathology	Main Findings
Lima et al. [45]	*Odoribacter splanchnicus*	Ulcerative Colitis	*Odoribacter splanchnicus* plays a crucial role in enhancing both metabolic functions and immune cell resilience against colitis.
Yang et al. [46]	*Lactobacillus acidophilus*, *coleohominis*, *gallinarum; Selenomonas artemidis*	Constipation, depression, and anxiety	Psychiatric symptoms were improved after the FMT.
Aggarwala et al. [47]	*Bacteroides vulgatus*, *uniformis ovatus*, *cellulosilyticus*; *Parabacteroides distasonis*, *merdae*	*Clostridioides difficile* infection	Significantly predicted the clinical outcomes of the transplantation for up to five years.
Lee et al. [48]	*Bacteroidales*	Non specific intestinal disorders	The study effectively used genome-resolved metagenomics to track and identify bacterial strains that persist in FMT recipients, deepening insights into microbiota dynamics post-transplant.
Zhang et al. [49]	*Faecalibacterium*; *Eubacterium*; *Roseburia*	Inflammatory Bowel Disease	Symptoms were improved after FMT but outcomes are linked to gut microbiota and methodology variations, emphasizing the need for standardized research to improve FMT effectiveness through microbial and metabolite adjustments.

**Table 2:** Key Studies in the literature on fecal microbiota transplantation in pancreatic pathology.

Authors	Study Objective	Methodology	Main Findings
Yang et al. [72]	Examined the efficacy of fecal microbiota transplantation (FMT) in treating moderately severe acute pancreatitis complicated by severe *Clostridioides difficile* infection (CDI).	A case study of FMT application in patient with moderately severe acute pancreatitis and severe CDI.	FMT led to the resolution of diarrhea within five days with no adverse events reported.
He et al. [81]	Investigated the efficacy of the FMT in adolescent patients with type 1 diabetes (T1D).	The administration of one to three cycles of the FMT to adolescent T1D patients with the monitoring of clinical outcomes and adjustments in therapeutic regimen.	FMT protocols showed potential as a viable treatment for autoimmune T1D, with implications for improving patient quality of life.
Li Liu et al. [91]	Explored the impact of the gut microbiota and their metabolites on acute pancreatitis (AP).	The investigation of gut dysbiosis correction through normobiotic FMT in AP patients.	Normobiotic FMT corrected gut dysbiosis caused by AP, reducing its severity including mitochondrial dysfunction, oxidative stress, and inflammation.
Ding et al. [92]	Assessed the role of the FMT in managing acute pancreatitis.	Randomized controlled trial comparing FMT versus saline administration in AP patients.	No significant difference in gastrointestinal recovery observed between FMT and saline groups. Increased levels of D-lactate and IL-6 were noted with FMT, suggesting potential adverse effects on the gastrointestinal barrier.
De Groot et al. [96]	Explored the effect of the FMT on preserving endogenous insulin production in patients with recently diagnosed T1D.	The division of participants with recent T1D onset into groups receiving autologous or allogeneic FMTs.	A significant preservation of beta-cell function observed in the autologous FMT group over 12 months, linked to specific plasma metabolites.

## Data Availability

Not applicable.

## Abbreviations

FMT—fecal microbiota transplantation, CDI—Clostridioides difficile infection, PC—pancreatic cancer, PDAC—pancreatic ductal adenocarcinoma, AP—acute pancreatitis, SCFAs—short-chain fatty acids, MSAP—moderately severe acute pancreatitis, CP—chronic pancreatitis, PERT—pancreatic enzyme replacement therapy, PEI—exocrine pancreatic insufficiency, T1D—type 1 diabetes, EcN—*Escherichia coli* Nissle.
